# H3K27me1 is essential for MMP-9-dependent H3N-terminal tail proteolysis during osteoclastogenesis

**DOI:** 10.1186/s13072-018-0193-1

**Published:** 2018-05-28

**Authors:** Kyunghwan Kim, Yonghwan Shin, Jinman Kim, Tobias S. Ulmer, Woojin An

**Affiliations:** 1 Department of Biochemistry and Molecular Medicine, Norris Comprehensive Cancer Center, University of Southern California, Los Angeles, CA 90089 USA; 2 Department of Physiology and Neuroscience, Zilkha Neurogenetic Institute, University of Southern California, Los Angeles, CA 90089 USA; 3 Department of Biology, College of Natural Sciences, Chungbuk National University, Cheongju, Chungbuk 361-763 Republic of Korea

**Keywords:** MMP-9, G9a, H3 proteolysis, H3K27me1, Osteoclast differentiation

## Abstract

**Background:**

MMP-9 plays a direct role in the activation of pro-osteoclastogenic genes by cleaving histone H3N-terminal tail (H3NT) and altering chromatin architecture. Although H3 acetylation at K18 has been shown to stimulate MMP-9 enzymatic activity toward H3NT, nothing is known about the influence of other H3NT modifications on this epigenetic reaction.

**Results:**

We show that H3 monomethylation at lysine 27 (H3K27me1) is essential for MMP-9-dependent H3NT proteolysis during RANKL-induced osteoclast differentiation. Through the recognition of H3K27me1 mark, MMP-9 localizes and generates H3NT proteolysis at the genes encoding osteoclast differentiation factors. By using RNAi and small molecule inhibitor approaches, we also confirmed that G9a is the major methyltransferase to catalyze H3K27me1 for MMP-9-dependent H3NT proteolysis and trigger the expression of osteoclast-specific genes.

**Conclusions:**

Our data establish new functions for G9a-mediated H3K27me1 in MMP-9-dependent H3NT proteolysis and demonstrate how histone modification can be exploited to regulate osteoclastogenic gene expression at the molecular level. Further studies are warranted to investigate the detailed mechanism by which G9a overexpression with concomitant dysregulation of osteoclastogenesis contributes to the pathogenesis of bone disorders.

**Electronic supplementary material:**

The online version of this article (10.1186/s13072-018-0193-1) contains supplementary material, which is available to authorized users.

## Background

Bone is a highly dynamic organ that is continuously remodeled by coordinated activities of two cell types, osteoclasts and osteoblasts [1, 2]. Osteoblasts are mononucleated mesenchymal stem cells that form bone matrix, whereas osteoclasts are bone-resorbing multinucleated cells that differentiate from hematopoietic progenitors of the myeloid lineage [3–5]. Osteoclast differentiation is induced by receptor activator of NF-κB ligand (RANKL), which is expressed as a membrane-bound protein in osteoblasts and provides osteoclast-specific differentiation signals [6, 7]. The binding of RANKL to its cognate receptor RANK on pre-osteoclast cell membrane stimulates the expression of key determinants of osteoclast differentiation such as NF-κB, c-Fos, and NFATc1 at the early stage of the process [1, 8]. These factors then initiate multiple signal transduction pathways to turn on downstream genes and activate quiescent osteoclast precursor (OCP) cells to become mature osteoclasts [1, 8]. The excessive formation and activity of osteoclasts lead to pathological bone diseases such as osteoporosis, rheumatoid arthritis, and tumor bone metastases [9, 10].

As for other eukaryotic genes, osteoclastogenic gene expression occurs in the context of chromatin, where DNA is wound around histone proteins to form chromatin structure [11, 12]. An essential step for understanding gene regulatory pathways at key differentiation time points, therefore, should lie in characterizing the enzymes responsible for reorganizing and potentiating particular chromatin domains. Not much progress has been made in supporting this idea, but there is some indirect evidence functionally linking chromatin modification to osteoclastogenic gene transcription. For example, the repressive histone mark H3K27me3 is removed from the master osteoclastogenic gene NFATc1 [13], while it is deposited into the anti-osteoclastogenic gene IRF8 [14]. Histone deacetylase inhibitors have antagonistic effects on osteoclast differentiation through the inactivation of NF-κB signaling pathways [6, 15], suggesting a potential role for histone acetylation in osteoclastogenic gene expression. Nonetheless, which factors are mainly responsible for establishing transcriptionally competent chromatin states and exactly how altered chromatin states trigger osteoclastogenic gene transcription remain poorly understood. Another large gap in our understanding of osteoclastogenic transcription program is the identification of chromatin factors that have the potential to link specific aspects of chromatin function to osteoclast differentiation processes.

Matrix metalloproteinases (MMPs) are a large family of extracellular enzymes, which function to remodel the pericellular environment, primarily through the cleavage of extracellular matrix proteins [16–18]. MMP-9, of special interest here, is a member of the MMP family and contains several conserved domains such as propeptide domain, catalytic domain with Zn^2+^-binding site, and hemopexin-like domain [17]. Like other MMP family members, MMP-9 is first synthesized as an inactive/latent 92-kDa proenzyme and subsequently converted to a fully active 82-kDa form by removal of the N-terminal propeptide domain [19]. Regarding MMP-9 functions, MMP-9 has been characterized as a major endopeptidase with an ability to degrade extracellular matrix and stimulate osteoclastogenesis [19]. Unexpectedly, however, our recent study revealed the nuclear function of MMP-9 as a protease that cleaves the histone H3N-terminal tail (H3NT) during osteoclast differentiation [20]. In strong support of these observations, our subcellular localization analysis by Western blotting, gelatin zymography, and immunofluorescence clearly demonstrated the nuclear translocation and accumulation of MMP-9 during RANKL-induced formation of mature osteoclasts. A functional role for the observed H3NT proteolysis in osteoclastogenic gene transcription was evident from our analysis of MMP-9-depleted OCP cells that had undetectable levels of H3NT proteolysis and OCP cell differentiation [20]. Our report also showed that MMP-9 enzymatic activity toward H3NT is significantly augmented by H3K18 acetylation (H3K18ac) and that p300/CBP is responsible for H3K18ac observed in OCP cells [20]. This is an important observation, meaning that p300/CBP-mediated H3K18ac has a functional role in osteoclastogenic gene expression and MMP-9 transactivation potential in OCP-induced cells.

In this study, we demonstrate that H3K27 monomethylation (H3K27me1) is necessary for the localization and function of MMP-9 at osteoclastogenic genes as a protease catalyzing H3NT proteolysis. The observed H3K27me1 is attributed to G9a and stabilizes the interaction of MMP-9 with nucleosomes. Supporting these data, selective inhibition of G9a-mediated H3K27me1 resulted in complete abrogation of RANKL-induced osteoclast formation and gene transcription. Therefore, G9a is a previously unrecognized regulator of osteoclast differentiation with a unique function relevant to MMP-9-dependent H3NT proteolysis.

## Results

### H3K27me1 is necessary for MMP-9-dependent H3NT proteolysis of nucleosome substrates

**Fig. 1:**
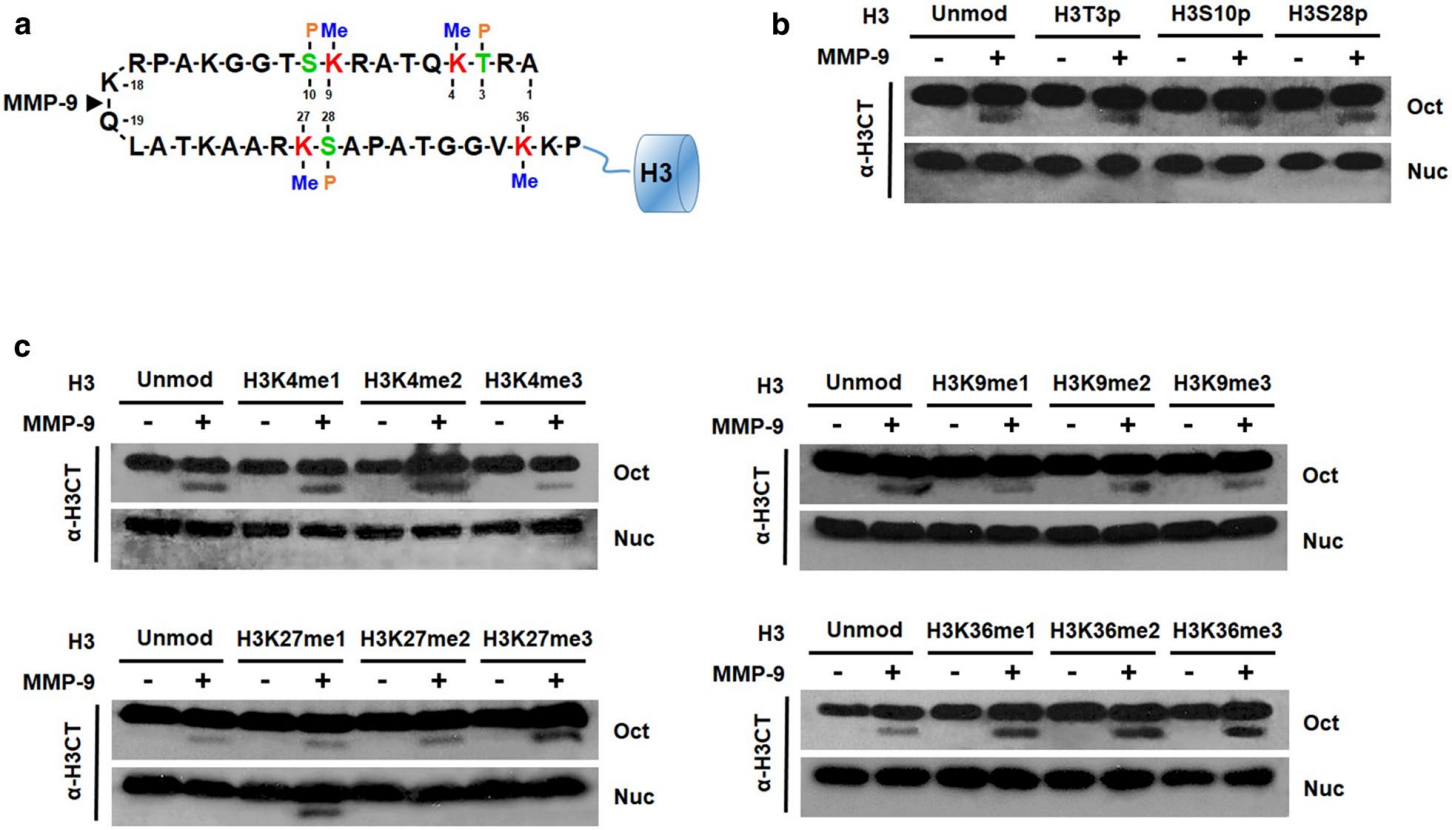
Requirement of H3K27me1 for MMP-9-dependent H3NT proteolysis. **a** Amino acid sequence of N-terminal tail (NT) of histone H3. Amino acids that can be methylated (me) or phosphorylated (p) are indicated. **b** In vitro H3NT cleavage assays were performed using recombinant histone octamer or reconstituted nucleosome array substrates unmodified or phosphorylated at H3T3p, H3S10p, or H3S28p. The extent of H3NT proteolysis was analyzed by Western blot with H3 C-terminal tail (CT) antibody. **c** As for **b** but using free histone octamers or nucleosome arrays methylated at H3K4, H3K9, H3K27, or H3K36

We have recently demonstrated that p300/CBP-mediated H3K18ac neutralizes the charged lysine residue at the primary cleavage site (P1) of MMP-9 and amplifies MMP-9 enzymatic activity toward both free and nucleosomal H3 substrates [20]. Since H3NTs are also subject to other types of modifications such as methylation and phosphorylation, investigating their possible effects on MMP-9-dependent H3NT proteolysis should be a logical extension of our study (Fig. 1a). Toward this end, we prepared recombinant histone octamers containing H3 analogs that are mono-, di-, or tri-methylated (me1, me2, or me3) at K4, K9, K27, or K36 and phosphorylated (p) at T3, S10, or S28. These histone octamers were then used to reconstitute differentially methylated or phosphorylated nucleosome arrays on a tandem DNA array containing seven copies of a 207-bp 601 nucleosome positioning sequence. In our initial H3NT proteolysis assays with histone octamers containing methylated H3 as substrates, an H3 C-terminal antibody detected a faster-migrating band representing H3NT-cleaved product in all reactions (Fig. 1c, Oct). It was apparent in these experiments that cleavage levels of methylated H3 were comparable with those of unmodified H3. Similarly, phosphorylation of H3 at T3, S10, or S28 showed no effects on MMP-9-dependent H3NT proteolysis in identical assays (Fig. 1b, Oct). When we extended in vitro cleavage assays to reconstituted nucleosome arrays, MMP-9 was unable to proteolyze H3NT in nucleosome array substrates carrying H3K4me1/me2/me3, H3K9me1/me2/me3, H3K27me2/me3, or H3K36me1/me2/me3 (Fig. 1c, Nuc). H3T3p, H3S10p, and H3S28p also had no effect on MMP-9-dependent H3NT proteolysis in our assays (Fig. 1b, Nuc). In sharp contrast, however, MMP-9 generated a reproducible H3NT proteolysis within H3K27me1 nucleosome arrays (Fig. 1c, Nuc). The observed effects are specific for nucleosome substrates, since H3K27me1 did not show a corresponding effect on free H3NT cleavage by MMP-9 in our assays (Fig. 1c, Oct). Based on these data, we concluded that H3K27me1 is required for MMP-9 to cleave H3NT in a nucleosome context.

### G9a catalyzes H3K27me1 and facilitates H3NT proteolysis during osteoclastogenesis

**Fig. 2:**
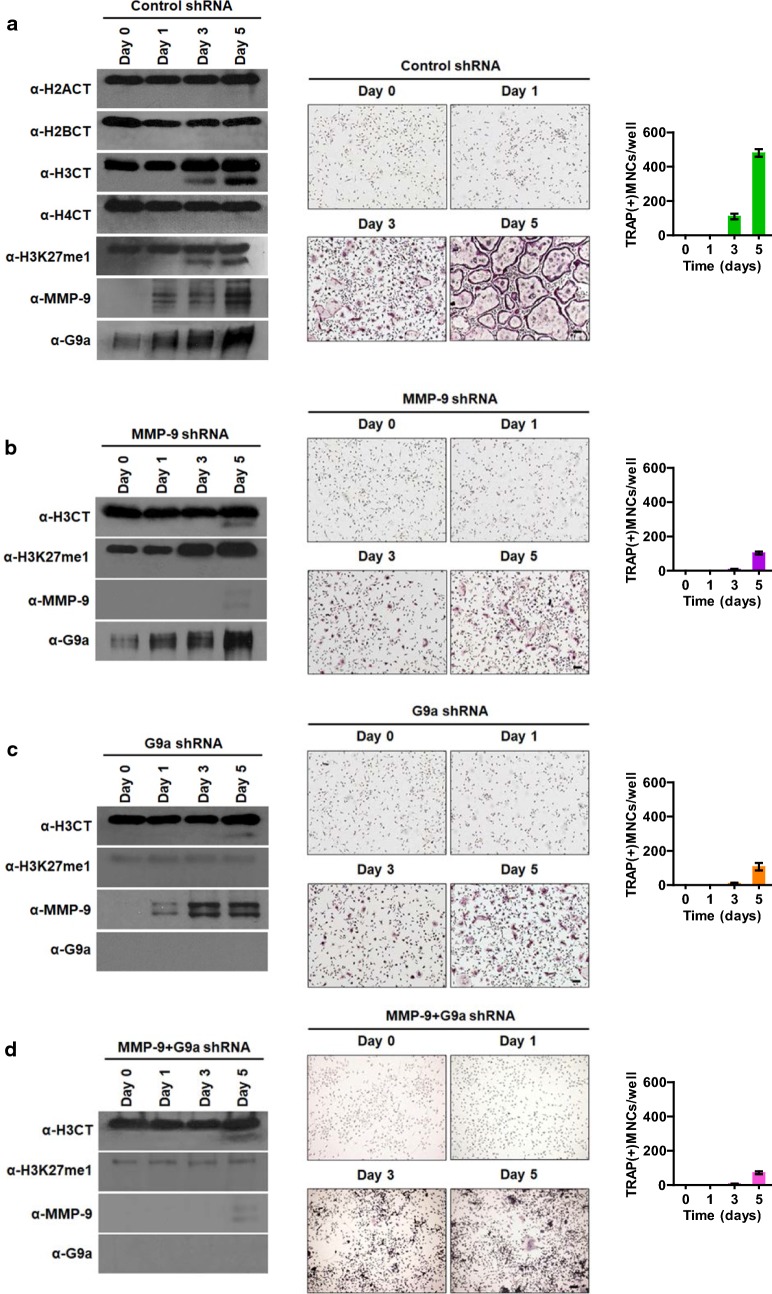
Dependence of osteoclastogenic H3NT proteolysis and H3K27me1 on G9a. **a** Chromatin was extracted from primary OCP cells treated with RANKL for 0, 1, 3, and 5 days and analyzed by Western blotting with H2A, H2B, H3, and H4 CT antibodies (left panel). OCP-induced cells were also fixed with formaldehyde, stained for TRAP (tartrate-resistant acid phosphatase), and photographed under a light microscope (10×) (middle panel). TRAP-positive cells containing three or more nuclei were counted as osteoclasts at the indicated days (right panel). **b** As for **a** but using OCP-induced cells depleted of MMP-9. **c** As for **a** but using OCP-induced cells depleted of G9a. **d** As for **a** but using OCP-induced cells depleted of both MMP-9 and G9a

We next wanted to examine whether H3K27me1 is similarly required for H3NT proteolysis during osteoclastogenesis and, if so, which histone methyltransferase (HMT) is responsible for H3K27me1. In our assay system, osteoclast precursor (OCP) cells are synchronously differentiated into TRAP-positive multinuclear osteoclasts in α-minimal essential medium (α-MEM) following RANKL treatment for 0, 1, 3, or 5 days. In agreement with our published data, we detected a fast-migrating H3 band representing H3NT proteolysis and an increase in MMP-9 expression after RANKL treatment (Fig. 2a). The loss of H3NT proteolysis following MMP-9 knockdown is also consistent with our previous demonstration of MMP-9-dependent H3NT proteolysis during osteoclast differentiation (Fig. 2b) [20]. In parallel experiments in which changes in H3K27me1 were analyzed over the same time period, a progressive increase in overall H3K27me1 levels was also evident (Fig. 2a, b), suggesting its contribution to H3NT cleavage process. To confirm the significance of H3K27me1 with respect to H3NT proteolysis more directly, we also transfected OCP-induced cells with plasmids expressing FLAG wild-type or K27-mutated H3 and prepared mononucleosomes as summarized in Additional file 1: Fig. S1. Mononucleosomes containing ectopic H3 were then purified by immunoprecipitations using anti-FLAG antibody. Our examination of purified mononucleosomes by Western blot detected a high level of H3NT cleavage in wild-type H3 nucleosomes, but the observed proteolysis was impaired in H3K27-mutated nucleosomes (Additional file 2: Fig. S2). These data indicate strongly that H3NT proteolysis in osteoclast differentiation pathway is dependent on H3K27me1.

To further investigate the role of H3K27me1 in the formation of mature osteoclasts, it was important to identify the HMT mediating the observed H3K27me1. For this objective, we suppressed the expression of three HMTs, EZH1, EZH2, and G9a, that were shown to catalyze H3K27me1 [21–25] (Fig. 2c and Additional file 3: Fig. S3). When OCP cells were depleted of EZH1 or EZH2, no obvious changes in H3K27me1 and H3NT proteolysis were detected in our Western blot analysis of chromatin fraction from RANKL-induced OCP cells (Additional file 4: Fig. S4). Because knockdown of EZH1 and EZH2 reduced levels of H3K27me2 and H3K27me3 (Additional file 4: Fig. S4), these results also indicate the indispensable role of H3K27me1 in osteoclastogenic H3NT proteolysis. On the contrary, specific knockdown of G9a in OCP-induced cells efficiently blocked H3K27me1 and almost completely abrogated H3NT proteolysis in our assays (Fig. 2c). Since G9a knockdown also decreased the average number of mature osteoclasts (Fig. 2c), these results confirm the functional contribution of G9a to osteoclast formation. Considering the possibility that G9a could promote osteoclast differentiation independently of MMP-9, we also checked whether double knockdown of G9a and MMP-9 exhibits more severe defects in osteoclastogenesis. Interestingly, however, the simultaneous knockdown of G9a and MMP-9 attenuated osteoclast differentiation in similar level as that observed in individual knockdown of G9a and MMP-9 (Fig. 2d). These data point to the dependence of pro-osteoclastogenic function of MMP-9 on G9a-mediated H3K27me1 and constitute a powerful argument that both MMP-9 and G9a are essential for efficient osteoclastogenesis.

**Fig. 3:**
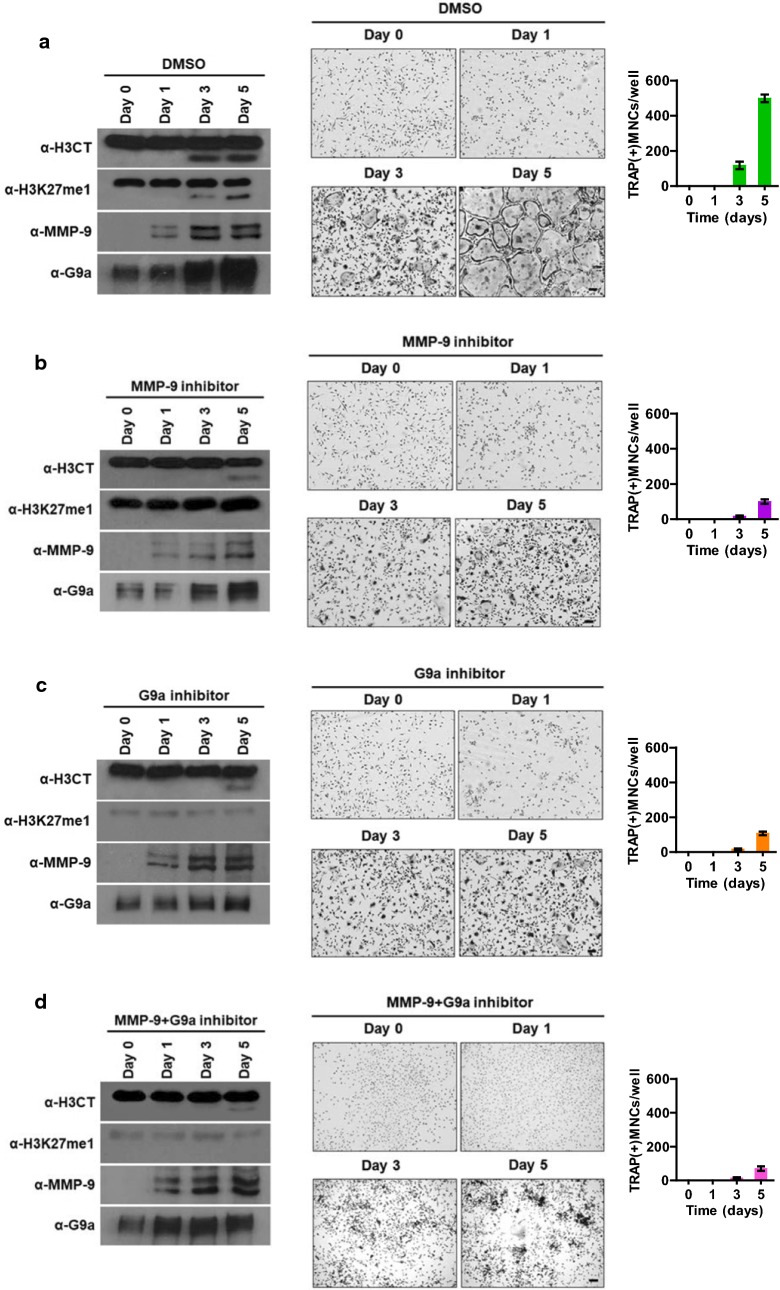
Abolishment of osteoclastogenic H3NT proteolysis and H3K27me1 by G9a inhibitor. OCP cells were treated with DMSO control (**a**), MMP-9 inhibitor (**b**), G9a inhibitor (**c**), or MMP-9 + G9a inhibitors (**d**) and cultured for 0, 1, 3, and 5 days with RANKL. Chromatin was isolated for Western blot analysis to assess H3NT proteolysis (left panel). Cells were also TRAP-stained middle panel) and counted (right panel) as in Fig. 2

In an attempt to support our knockdown data, we also tested whether chemical inhibition of G9a, EZH1, and EZH2 would affect H3K27me1 and H3NT proteolysis by using two inhibitors, BIX01294 and UNC1999. Of note, BIX01294 has an inhibitory property for the methyltransferase activity of G9a [26], whereas UNC1999 inhibits the enzymatic activities of both EZH1 and EZH2 [27]. In analyzing the effects of EZH1/2 inhibitor UNC1999, we detected lower levels of H3K27me2 and H3K27me3, but failed to see any apparent changes in MMP-9 protease activity toward H3NT (Additional file 5: Fig. S5). Meanwhile, treatment of OCP-induced cells with 1.5 µM G9a inhibitor BIX01294 led to a pronounced inhibition of H3NT proteolysis and osteoclast development (Fig. 3c). Consistent with our published data [20], a significant decrease in H3NT proteolysis was also observed upon treatment with 10 nM MMP-9 inhibitor I. Moreover, a failure to generate higher levels of H3NT proteolysis and osteoclastogenesis upon treatment with both G9a inhibitor BIX01294 and MMP-9 inhibitor I, compared to the levels generated by their individual treatment, confirmed again the dual requirement of G9a and MMP-9 for signal transduction pathways operating in osteoclast differentiation (Fig. 3b, d).

### G9a-mediated H3K27me1 is crucial for MMP-9 recruitment and function at target genes

Having established the requirement of G9a-mediated H3K27me1 for MMP-9-dependent H3NT proteolysis and proficient osteoclast differentiation, we next sought to explore whether G9a is also directly involved in the expression of MMP-9 target genes. We recently developed a technique called ChIP of acetylated chromatin (ChIPac) (schematized in Additional file 6: Fig. S6) and demonstrated that H3NT proteolysis is associated with RANKL-induced activation of genes necessary for osteoclast differentiation [20]. In this new method, we made use of methylene blue to cross-link chromatin and acetic anhydride to completely acetylate all lysine residues in fragmented chromatin. H3K14ac-specific antibody was then used to selectively precipitate intact H3NT-containing chromatin, and a reduced PCR or sequencing signal intensity relative to the control ChIPac reactions using an H3CT antibody is indicative of osteoclastogenic H3NT proteolysis. During the process of osteoclast formation, MMP-9 was shown to generate H3NT cleavage in promoter, coding region, or both for target gene transcription in a gene-specific manner [20]. Thus, we examined the localization of G9a at Nfatc1, Lif, and Xpr1 genes representing each H3NT-cleaved group in OCP-induced cells by ChIPac-qPCR, as described recently [20]. Two sets of primers were used to detect MMP-9, G9a, and H3K27me1 in the promoter (P) and coding region (CR) by qPCR.

**Fig. 4:**
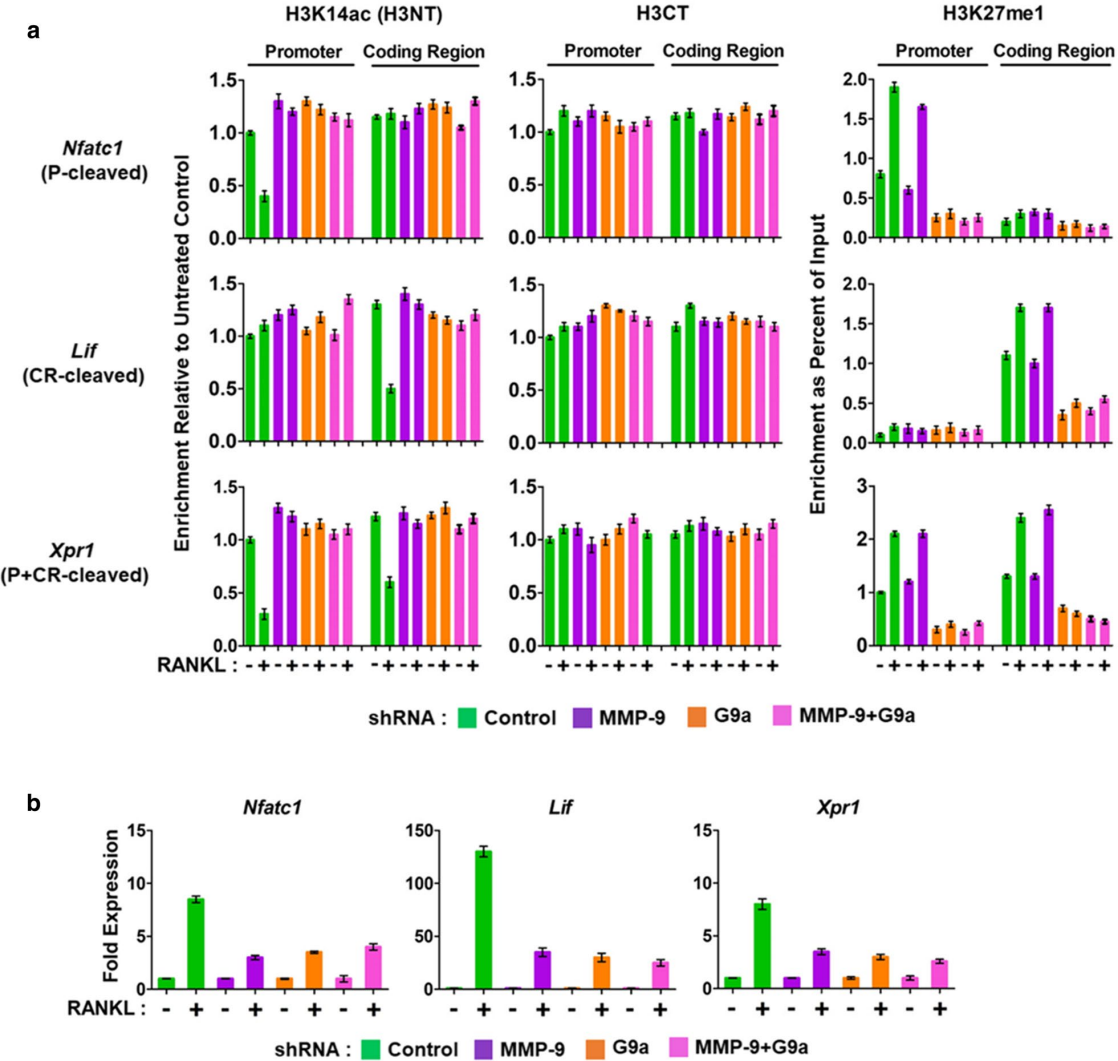
Impaired H3NT proteolysis and osteoclastogenic gene transcription in G9a-depleted OCP cells. **a** Control, MMP-9-, G9a-, or MMP-9 + G9a-depleted OCP cells were cultured with RANKL to induce osteoclastogenesis for 3 days, and ChIPac was performed using H3K14ac (H3NT), H3CT, and H3K27me1 antibodies. H3NT cleavage levels were determined at the promoter (P) and coding regions (CR) of Nfatc1 (P-cleaved), Lif (CR-cleaved), and Xpr1 (P + CR-cleaved) genes by qPCR with primers used in our previous study [20] and are listed in “Methods” section. **b** RT-qPCR assays were performed to determine fold changes in Nfatc1, Lif, and Xpr1 expression in 3-day RANKL-induced OCP cells depleted of MMP-9 and/or G9a

**Fig. 5:**
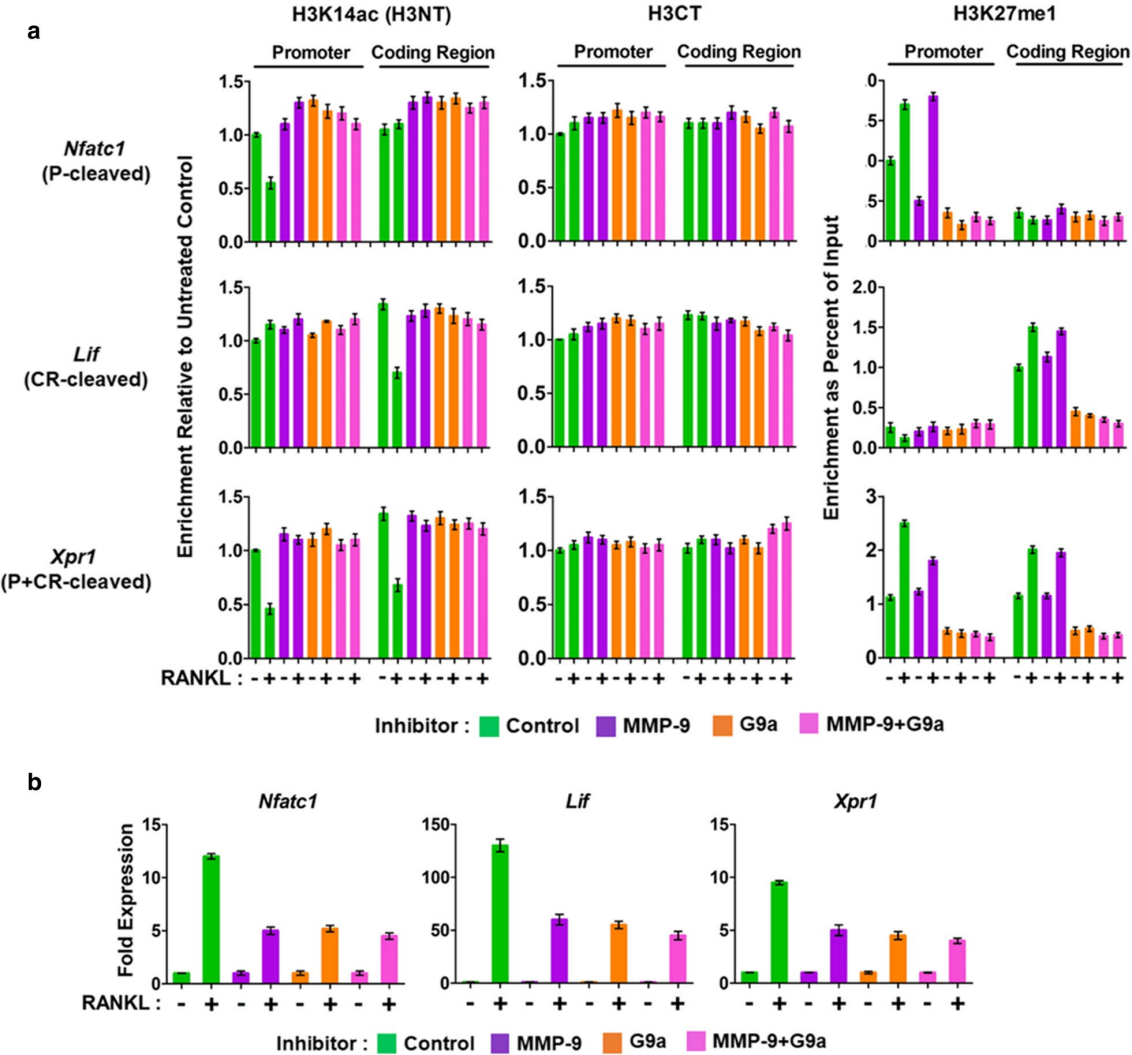
Impaired H3NT proteolysis and osteoclastogenic gene transcription in G9a inhibitor-treated OCP cells. **a** ChIPac-qPCR assays were conducted to assess H3NT proteolysis levels in 3-day OCP-induced cells treated with MMP-9 and/or G9a inhibitors. **b** RT-qPCR to quantitate Nfatc1, Lif, and Xpr1 transcript levels in 3-day OCP-induced cells treated with MMP-9 and/or G9a inhibitors

Consistent with our recently published data [20], RANKL treatment of OCP cells resulted in a rapid accumulation of MMP-9 in the P, CR, and both regions of Nfatc1, Lif, and Xpr1 genes, respectively (Fig. 4a). The localization patterns of MMP-9 were similar to those observed for H3K27me1 in these target genes, implicating H3K27me1 as the major recruitment signal for MMP-9. H3K27me1 levels were reduced at the target genes after G9a knockdown, and such changes diminished the measured levels of P and CR occupied by MMP-9 (Fig. 4a). No detectable effects of MMP-9 knockdown on G9a occupancy at the target genes indicate that MMP-9 is dispensable for G9a recruitment and function. To further confirm the results, the ChIPac-qPCR assays were repeated using OCP-induced cells treated with MMP-9 and G9a inhibitors. As was observed with G9a knockdown, OCP-induced cells treated with G9a inhibitor show significantly lower levels of H3K27me1 and MMP-9 at the target genes (Fig. 5a). Expectedly, neither G9a localization nor H3K27me1 at the target genes was affected upon treatment with MMP-9 inhibitor (Fig. 5a).

Given the demonstrated reliance of MMP-9 on G9a-mediated H3K27me1 for its target gene localization, we also examined the role of G9a with respect to RANKL-induced expression of osteoclast-specific genes. Our RT-qPCR analysis showed that G9a depletion in OCP-induced cells caused three- to sevenfold decreases in mRNA levels of Nfatc1, Lif, and Xpr1 genes (Fig. 4b). Congruent with these data, treatment of OCP cells with G9a inhibitor also led to impaired expression of the target genes upon RANKL-induced differentiation (Fig. 5b). In addition, when OCP cells were depleted of both G9a and MMP-9 or treated with G9a and MMP-9 inhibitors together, target gene transcription was repressed, but the level of repression was similar to that detected in knockdown or inhibition of G9a or MMP-9, again pointing to G9a-mediated H3K27me1 as an essential epigenetic mark for MMP-9 recruitment and function (Figs. 4b, 5b). Together, these data support the direct function of G9a in regulating MMP-9 target gene pathways necessary for proficient osteoclast differentiation.

### MMP-9 specifically binds to H3K27me1 nucleosomes

**Fig. 6:**
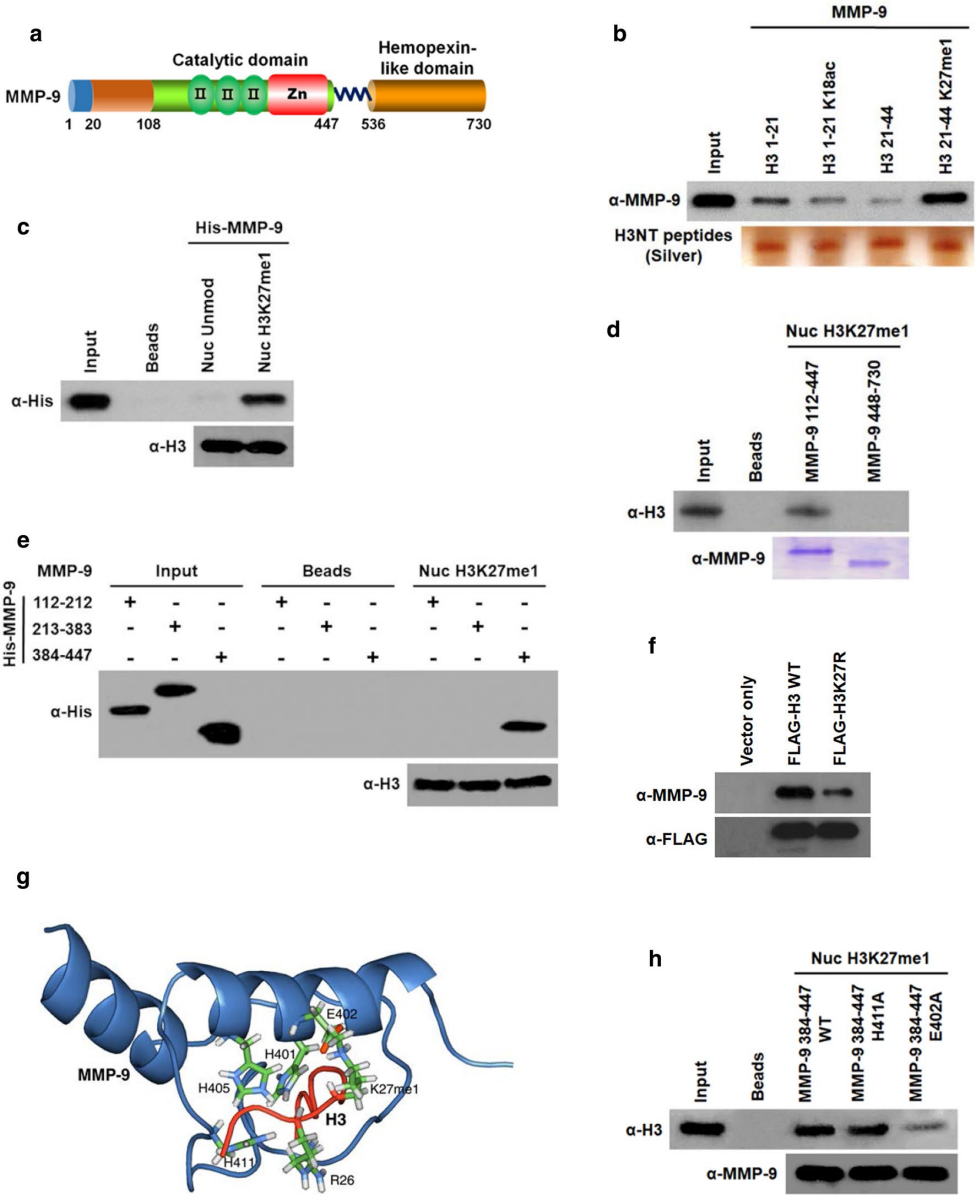
MMP-9 binding to H3K27me1 nucleosomes. **a** Schematic depiction of the domain structure of MMP-9. **b** Peptide pull-down assays with biotinylated H3 1–21 and 21–44 peptides and recombinant His-MMP-9 were analyzed by Western blotting with anti-His antibody. H3 peptides were unmodified, K18ac or K27me1 as indicated. Lane 1 represents 10% of the input MMP-9. **c** Nucleosomes were reconstituted on a 207-bp 601 nucleosome positioning sequence using unmodified or H3K27me1 histone octamers and immobilized on streptavidin beads. His-MMP-9 was incubated with immobilized nucleosomes, and its binding to nucleosomes was analyzed by Western blotting with anti-His antibody. Lane 1 contains 10% of the input MMP-9. **d** H3K27me1 nucleosomes were incubated with immobilized MMP-9 N-terminal (amino acids 112–447) and C-terminal (amino acids 448–730) domains. After extensive washing, the binding of H3K27me1 nucleosomes to MMP-9 domains was determined by Western blotting with anti-H3 antibody. Input corresponds to 10% of H3K27me1 nucleosomes used in the binding reactions. **e** After incubation with H3K27me1 nucleosomes, the binding of MMP-9N-terminal subregions to nucleosomes was determined by Western blotting with anti-His antibody. Input lanes 1–3 represent 10% of MMP-9 fragments used in the binding reactions. **f** OCP-induced cells were transfected with FLAG-H3 wild type (WT) or K27R mutant (K27R), and mononucleosomes were prepared by micrococcal nuclease digestion as summarized in Figure S3. Mononucleosomes containing ectopic H3 were immunoprecipitated from total mononucleosomes with FLAG antibody and analyzed by Western blotting with anti-MMP-9 antibody. **g** Model of the MMP-9-H3K27me1 interaction. PDB entries 4h3x (mMMP-9) and 3avr (H3.1) were used in docking simulations using the program Cluspro 2.0 [28–30]. Simulations were run with non-methylated H3. For context, H3K27 is shown monomethylated. **h** Nucleosome binding assays were conducted as in **e**, except that His-MMP-9 amino acids 384–447 carrying E402A mutation were used

The results of the above experiments argue persuasively that H3K27me1 is indispensable for the stable localization and function of MMP-9 at target genes. However, it is not clear whether the observed effects of H3K27me1 reflect its role as a docking site to facilitate the recruitment of MMP-9 to target genes. To check this possibility, H3NT unmodified or K27me1 peptides corresponding to amino acids 1–21 or 21–44 were immobilized on streptavidin-coated wells and monitored the binding for MMP-9. Our results showed that MMP-9 strongly binds to H3NT K27me1 peptides, whereas other H3NT peptides displayed only weak interaction with MMP-9 (Fig. 6b). To further confirm these results, we reconstituted nucleosomes containing H3 unmodified or K27me1 on a 601 nucleosome positioning sequence and checked the binding of MMP-9. In agreement with the interactions observed with H3NT peptides, we detected a remarkable binding preference of MMP-9 for the immobilized nucleosome containing H3K27me1 over the nucleosome containing H3 unmodified (Fig. 6c). Additionally, in mapping the interaction region of mature MMP-9, we found that N-terminal region (amino acids 112–447) of MMP-9 retained strong affinity for H3K27me1 nucleosome, whereas no apparent interaction was observed with the remainder (amino acids 448–730) of the protein (Fig. 6a, d). Also, in similar binding experiments using three N-terminal subregions, MMP-9 amino acids 384–447 directly interacted with H3K27me1 nucleosome, but MMP-9 amino acids 112–212 and 213–383 failed to show any detectable interaction under the same conditions (Fig. 6e).

In order to gain more support for the in vitro binding results above, OCP cells were transfected with expression vectors for FLAG-H3 wild type or K27R mutant. After treating OCP cells with RANKL for 3 days, soluble chromatin was prepared from cell nuclei and digested with micrococcal nuclease to yield mainly mononucleosomes. Mononucleosomes containing ectopic H3 wild type or K27R mutant were then specifically immunoprecipitated with anti-FLAG antibody. When we analyzed the association of endogenous MMP-9 with these purified nucleosomes, we found that MMP-9 bound avidly to wild-type H3 nucleosomes, but bound minimally to K27R-mutated H3 nucleosomes (Fig. 6f). To identify amino acid residues critical for the MMP-9-H3NTK27me1 interaction, we next generated a structural model of the MMP-9-H3NTK27me1 complex based on existing crystal structures using the program Cluspro 2.0 [28–30] (Fig. 6g). The model identified E402 of MMP-9 to contact anionic residues in H3NTK27me1. Moreover, the Zn^2+^-binding site of MMP-9 was suggested to interact with H3NTK27me1. Consistent with this model, in vitro binding assays testing the E402A substitution showed a diminished binding of MMP-9 to H3NTK27me1, supporting a role for electrostatic contacts involving this residue in the interaction (Fig. 6h). In contrast, the H411A substitution was inconspicuous, which leaves the role of the Zn^2+^-binding site ambiguous (Fig. 6h). As judged by crystal structures of methyltransferases and demethylases [28, 30–32], the differentiation of H3K27 methylation states by MMP-9 likely arises from steric and electrostatic differences between different methylation levels. Together, these results constitute a powerful argument that, although other factors and signals may be involved, the initial recruitment of MMP-9 at target genes is dependent on the recognition of H3NTK27me1 through the N-terminal domain of MMP-9.

## Discussion

There has been a growing interest in understanding how epigenetic processes regulate gene transcription at various stages of osteoclast differentiation, activation, and survival. MMP-9 is highly expressed in OCP cells and is known to play a key role in RANKL-induced osteoclast differentiation. The general view of MMP-9 function in osteoclastogenesis is that MMP-9 digests structural components of extracellular matrix and cellular surface facilitating cell migration and adhesion [16]. However, this common idea has been changed recently by our report documenting that, beside regulating extracellular matrix remodeling, MMP-9 moves into the nucleus and generates active transcription states of osteoclastogenic genes by proteolytically cleaving H3NT at promoter and coding regions [20]. Also, our study provided the first evidence that p300/CBP-mediated H3K18ac is required for MMP-9 to mediate H3NT proteolysis and active expression of genes encoding positive regulators of osteoclastogenesis [20]. Since other histone modifications can also affect gene expression, one obvious gap in our understanding of MMP-9-regulated transcription mechanism lies in the identification of additional histone marks that may be involved in MMP-9 activity toward H3NT.

**Fig. 7:**
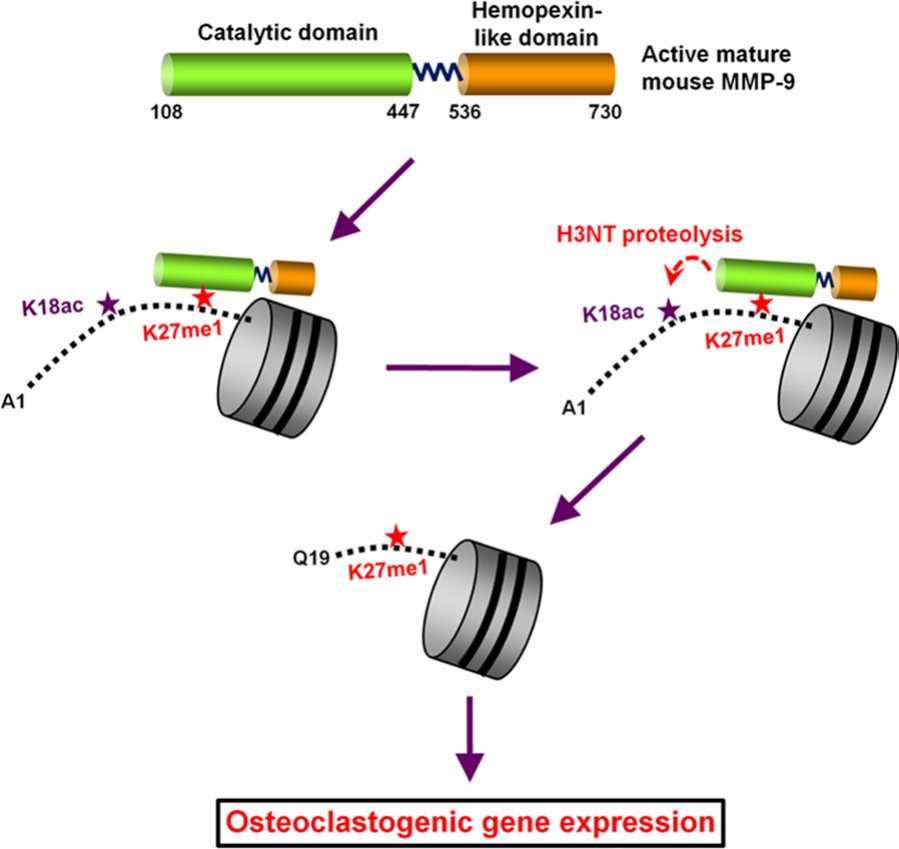
Working model. Our previous study revealed that MMP-9 catalyzes H3NT proteolysis for osteoclastogenic gene activation and that CBP/p300-mediated H3K18ac facilitates MMP-9 protease activity. Here, we depict that G9a-mediated H3K27me1 is necessary for MMP-9-dependent H3NT proteolysis in chromatin and proficient osteoclast differentiation. Without G9a-mediated H3K27me1, MMP-9 cannot localize at osteoclastogenic genes and mediate H3NT proteolysis. In the presence of G9a-mediated H3K27me1, MMP-9 gets recruited to target loci, facilitating H3NT proteolysis, leading to RANKL-induced gene transcription, and promoting osteoclast differentiation

In the present study, we focused on potential roles of H3 methylation and phosphorylation in MMP-9-dependent H3NT proteolysis. Unexpectedly, our in vitro cleavage assays with nucleosome arrays containing differentially modified H3 revealed that H3K27me1 is necessary for H3NT proteolytic activity of MMP-9. Our observation that MMP-9 can efficiently cleave free H3 substrates regardless of their H3K27me1 states is supportive of the idea that H3K27me1 specifically enhances MMP-9 enzymatic activity toward H3NT in a nucleosome context. We extended these in vitro findings in subsequent cellular studies revealing that MMP-9 protease activity toward H3NT is dependent on G9a-mediated H3K27me1 during RANKL-induced osteoclast formation. Notably, data presented here also indicate that G9a levels are highly elevated in response to RANKL treatment, leading to a sharp increase in H3K27me1. Moreover, the level of G9a-mediated H3K27me1 correlates directly with the extent of MMP-9-dependent H3NT proteolysis and osteoclastogenesis. This finding underscores the importance of H3K27me1 in accurate targeting of MMP-9 within defined chromatin regions, and taken together with our recently described H3K18ac-augmented MMP-9 clipping activity, it also suggests that these epigenetic alterations in H3NT directly influence MMP-9-dependent expression of pro-osteoclastogenic genes (Fig. 7).

Even though EZH1 and EZH2 were shown to mediate H3K27me1 in vitro assays and in certain cell types [21–24], our knockdown data indicate that they do not participate in generating H3K27me1 and that G9a is mainly responsible for catalyzing this osteoclastogenic histone mark. Another argument in favor of G9a to act as the major H3K27me1 methyltransferase comes from the experiments showing that treating OCP cells with G9a inhibitors, but not with EZH1/EZH2 inhibitors, leads to near complete loss of H3K27me1 and H3NT proteolysis. This finding is consistent with a recent report that G9a inhibitor BIX01294 reduced RANKL-induced osteoclast formation from RAW 264.7 cells [33]. In this study, H3K9me was assumed to play a causal role for G9a stimulation of osteoclast differentiation, and BIX01294 treatment was considered to have inhibitory effects on H3K9me process. However, our data provide strong evidence for the significance of H3K27me1, rather than H3K9me, for the osteoclastogenic function of G9a. Also, G9a inhibitor-enhanced osteoclastogenesis intrinsically depends upon MMP-9 function as an H3NT protease, since knockdown of G9a in MMP-9-depleted OCP cells failed to generate more attenuation of osteoclastogenesis. In this regard, H3K27me1 should be recognized as an epigenetic mark reflecting the initiation and progression of osteoclastogenic process.

Further analysis of G9a-depleted OCP cells showed that G9a has a direct impact on the expression of osteoclastogenic genes and revealed an intriguing new transcription pathway regulating osteoclast differentiation. After identifying osteoclast gene transcription being up-regulated by G9a, we also realized that these genes were enriched by H3K27me1, MMP-9, and cleaved H3 species in a G9a-dependent manner. These results demonstrate the direct action of G9a on transcriptional program required for osteoclastogenesis and support the concept that G9a exerts a coactivation function in MMP-9-driven transactivation. Our ChIPac analyses showed that G9a generates H3K27me1 in promoter or coding regions or both regions of osteoclastogenic genes and that H3NT proteolysis patterns match well with H3K27me1 states. However, high levels of transcription were accomplished in all these cases. This observation suggests a function for G9a-mediated H3K27me1 at the initiation or elongation step of transcription in a gene-specific manner. It is also conceivable that the levels of H3NT proteolysis are not proportional to transcription rates and H3NT proteolysis targeted to certain regions is sufficient to induce gene transcription.

Toward understanding how H3K27me1 can facilitate MMP-9-dependent H3NT proteolysis, we demonstrate that H3K27me1 is essential for MMP-9 binding to nucleosomal H3NTs. Only when H3K27me1 was introduced into nucleosomes did it contribute to MMP-9 proteolytic activity. Thus, MMP-9 appears to utilize some specific structural features to recognize H3K27me1 nucleosomes in osteoclastogenic target genes (Fig. 7). Reciprocally, the results also suggest that H3K27me1 may serve principally as a mark for MMP-9 recruitment to target genes, with no additional role in MMP-9-dependent H3NT proteolysis. These observations are reminiscent of cathepsin L, of which capability to catalyze H3NT proteolysis was enhanced by H3K27me2 [34]. Nonetheless, no detectable effects of H3K27me2 in our assays suggest the presence of two different regulatory mechanisms involving H3K27me1 for MMP-9 and H3K27me2 for cathepsin L. Thus, in addition to its known contribution to gene transcription [21, 35], our results underscore the significance of H3K27me1 in helping MMP-9 target specific genes to mediate H3NT proteolysis. Defining the molecular basis for H3K27me1 effects on MMP-9 activity is beyond the scope of this first report but will be of interest for how epigenetic signals may alter intrinsic MMP-9 properties in OCP cells in response to RANKL stimulation. Based on the functional connection uncovered between G9a-mediated H3K27me1 and MMP-9-dependent H3NT proteolysis, we speculate that inventing strategies to block osteoclastogenic G9a methyltransferase activity could provide an effective treatment for bone loss diseases.

## Conclusions

Results presented here describe a role for G9a in MMP-9-dependent H3NT proteolysis and gene transcription by catalyzing H3K27me1 during RANKL-induced osteoclast differentiation.

These results establish a direct functional link between G9a and MMP-9 in the context of pro-osteoclastogenic transcriptional programs. Our model suggests that G9a-mediated H3K27me1 serves as an essential mark for MMP-9 recruitment and proteolytic activity at genes encoding positive regulators of osteoclast differentiation, thus keeping them active (Fig. 7).

## Methods

### Plasmid construction and materials

Core histones and MMP-9 proteins were expressed in *Escherichia coli* Rosetta 2 (DE3) pLysS cells (Novagen) and purified from inclusion bodies as described recently [20]. To generate mutant H3 and MMP-9 expression vectors, H3 and MMP-9 cDNAs were mutated by the QuikChange II site-directed mutagenesis kit (Agilent Technologies) before the construction. Further details of plasmid constructions are available upon request. G9a inhibitor BIX01294 is from Santa Cruz Biotech, and EZH1/2 inhibitor UNC1999 and MMP-9 Inhibitor I are from Sigma. Antibodies used in this study are as follows: H2A, H2B, H3, H4, and EZH2 antibodies from Abcam; H3K27me1 and EZH1 antibodies from Millipore; G9a, actin, and FLAG antibodies from Sigma; His antibody from Novagen; and MMP-9 antibody from Santa Cruz Biotech.

### In vitro H3NT cleavage assays

Recombinant histone octamers and nucleosome arrays containing unmodified, methylated, or phosphorylated H3 were prepared following the procedure described [20, 36]. MMP-9 was incubated with 1 µg of histone octamer or 2 µg of nucleosome arrays, and H3NT cleavage was determined by Western blotting with H3 C-terminal antibody [20].

### Osteoclast differentiation and H3NT cleavage analysis

Osteoclast precursor (OCP) cells were prepared as recently described [20]. To generate osteoclasts, OCP cells were cultured in the presence of 30 ng/ml macrophage colony-stimulating factor (M-CSF) and 50 ng/ml receptor activator of nuclear factor kappaB ligand (RANKL). On days 0, 1, 3, and 5, the cells were fixed with formaldehyde and stained for tartrate-resistant acid phosphatase (TRAP) using an acid phosphatase leukocyte kit (Sigma). TRAP-positive multinucleated cells containing three or more nuclei were counted as osteoclasts under a light microscope. In certain instances, media were supplemented with G9a inhibitor BIX01294 (1.5 µM), EZH1/2 inhibitor UNC1999 (2 µM), and MMP-9 inhibitor I (10 nM) to evaluate their effects on OCP cell differentiation. To determine the levels of H3NT proteolysis, nuclei were isolated from OCP-induced cells in buffer A (10 mM HEPES, pH 7.4, 10 mM KCl, 1.5 mM MgCl2, 0.34 M sucrose, 10% glycerol, 1 mM DTT, 5 mM β-glycerophosphate, 10 mM NaF, protease inhibitors, and 0.2% Triton X-100) and chromatin was extracted in buffer B (3 mM EDTA, 0.2 mM EGTA, 1 mM DTT, 5 mM β-glycerophosphate, 10 mM NaF, and protease inhibitors). Western blot analysis was performed using H3 C-terminal antibody as previously described [20].

### RNA interference, RT-qPCR, and ChIPac-qPCR

Lentiviral particles were generated in HEK-293T cells by co-transfecting plasmids encoding VSV-G, NL-BH, and pLKO.1-shRNA (Addgene) for MMP-9 (5′-GAGGCATACTTGTACCGCTAT-3) or G9a (5′-AGACATTTCTCCATCAGAGAC-3′). OCP cells were transduced with these viruses or 10 nM MMP-9-INI (Santa Cruz) for 3 days prior to differentiation. Total RNA was isolated from OCP-induced cells using the Qiagen RNeasy kit (Qiagen, Valencia, CA) and reverse-transcribed using the iScript cDNA synthesis kit (Bio-Rad) and PerfeCta SYBR Green FastMix (Quanta Biosciences). ChIPac-qPCR assays were performed using chromatin that was fixed with 10 µM methylene blue and acetylated with 20 mM acetic anhydride as described [20]. H3K14ac, H3CT, and H3K27me1 antibodies were used to immunoprecipitate cross-linked chromatin. The immunoprecipitated protein–DNA complexes were recovered, washed, and incubated overnight at 65 °C to reverse the cross-linking. DNA fragments were purified and analyzed with the primers that amplify the promoter (P) and coding regions (CR) of Nfatc1 (P-cleaved), Lif (CR-cleaved), and Xpr1 (P + CR-cleaved) genes. The sequences of primers used for qPCR are as follows: Nfatc1 (P: 5′-GAAGTGGTAGCCCACGTGAT-3′, 5′-TCTTGGCACCACATAAACCA-3′; CR: 5′-GGGTCAGTGTGACCGAAGAT-3′, 5′-GGAAGTCAGAAGTGGGTGGA-3′; mRNA: 5′-CTCGAAAGACAGCACTGGAGCAT-3′, 5′-CGGCTGCCTTCCGTCTCATAG-3′), Lif (P: 5′-CTCTGGCTGTCCTGGAACTC-3′, 5′-CCAGGACCAGGTGAAACACT-3′; CR: 5′-ATCTTGTGGCTTTGCCAACT-3′, 5′-AGTCCTTGCCTGTCTTTCCA-3′; mRNA: 5′-TACTGCTGCTGGTTCTGCAC-3′, 5′-TGAGCTGTGCCAGTTGATTC-3′), and Xpr1 (P: 5′-AGGACCTTCGGAAGAGCAGT-3′, 5′-CAGCAAGCAGCTCATAACCA-3′; CR: 5′-GGTGGGTTCCACTGAAAGAA-3′, 5′-GGTTCCTCTGACCAAAAGCA-3′; mRNA: 5′-AGGAGCGTGTCCAACATAGG-3′, 5′-CCACGAGATGTTTCCAGGAT-3′).

### H3 tail peptide and nucleosome binding assays

For H3NT peptide binding assays, biotinylated forms of H3NT peptides unmodified, acetylated at K18, or monomethylated at K27 (EZBiolab Inc) (2 μg) were immobilized on streptavidin–agarose beads. After washing with BC250/0.1% Nonidet P-40, His-MMP-9 was incubated with H3NT peptides-bound beads in BC200/0.1% Nonidet P-40 for 3 h at room temperature. After extensive washing with BC200/0.1% NP-40, MMP-9 interaction was analyzed by Western blotting with anti-His antibody. For nucleosome binding assay, H3 unmodified/H3K18ac/H3K27me1 nucleosomes were reconstituted by mixing recombinant histone octamers and biotinylated 207-bp 601 nucleosome positioning sequence templates at a ratio of 1:1.2 (w/w) and salt gradient dialysis and purified by sedimentation in a 5–30% (vol/vol) glycerol gradient as described previously [36]. Nucleosomes (1 μg) were immobilized on streptavidin–agarose beads (Novagen) and incubated with His-MMP-9 proteins for 16 h on ice. After washing with BC250/0.1% Nonidet P-40, bound MMP-9 proteins were detected by Western blotting.

### Nucleosome purification and analysis

OCP cells were transfected with expression vectors for H3 wild type or K27R mutant containing a C-terminal FLAG tag. After 3-day RANKL treatment, cells were harvested and lysed with buffer A (20 mM HEPES, pH 7.4, 10 mM KCl, 1.5 mM MgCl2, 0.34 M sucrose, 10% glycerol, 1 mM dithiothreitol, and protease inhibitor cocktail) containing 0.2% Triton X-100. Nuclei were pelleted by centrifugation at 1000*g*, resuspended in buffer A containing 2 mM CaCl_2_, and digested with 0.6 U micrococcal nuclease (Sigma) at 37 °C for 20 min. Digested nuclei were collected and incubated in nuclear extraction buffer (20 mM HEPES, pH 7.4, 420 mM NaCl, 1.5 mM MgCl_2_, 0.2 mM EGTA, and protease inhibitor cocktail) for 1 h and centrifuged to remove nuclear debris. After adjusting the salt concentration of the extract to 150 mM NaCl, ectopic H3-containing nucleosomes were isolated by immunoprecipitation using anti-FLAG M2 agarose beads in washing buffer (20 mM HEPES, pH 7.8, 300 mM NaCl, 1.5 mM MgCl_2_, 0.2 mM EGTA, 10% glycerol, 0.2% Triton X-100, and protease inhibitor cocktail). Levels of H3NT proteolysis of bead-bound nucleosomes were analyzed by Western blotting with anti-FLAG antibody. The purified nucleosomes were also subject to Western blotting with anti-MMP-9 antibody.

### Statistical analysis

 All quantitative data are presented as mean ± SD. Statistical analyses of datasets were performed with Student’s two-tailed *t* test or two-way ANOVA followed by Bonferroni’s comparison test. GraphPad Prism (GraphPad Software Inc.) was used for all analyses. A *P* value < 0.05 was considered statistically significant.

## Additional files

**Additional file 1: Fig. S1.** Workflow of the purification method used for isolation of ectopic H3 nucleosomes.

**Additional file 2: Fig. S2.** Abolishment of osteoclastogenic H3NT proteolysis by H3K27R mutation. Mononucleosomes containing ectopic H3 were purified from OCP-induced cells expressing H3 wild type or K27R mutant with C-terminal FLAG tag as summarized in Additional file 1: Fig. S1 and analyzed by Western blotting with anti-FLAG antibody.

**Additional file 3: Fig. S3.** Validation of specific knockdown of EZH1 and EZH2. OCP cells were transduced with lentiviral shRNAs targeting EZH1 (**a**) and EZH2 (**b**), and knockdown efficiency and specificity were determined by Western blot.

**Additional file 4: Fig. S4.** Determination of the effects of EZH1/EZH2 knockdown on osteoclastogenic H3NT proteolysis. **a** Chromatin was purified from mock-depleted, OCP-induced cells, and Western blot analysis for H3NT proteolysis was performed as described in Fig. 2. **b** As for (**a**) but using chromatin from EZH1-depleted, OCP-induced cells. **c** As for (**a**) but using chromatin from EZH2-depleted, OCP-induced cells.

**Additional file 5: Fig. S5.** Determination of the effects of EZH1/EZH2 inhibitors on osteoclastogenic H3NT proteolysis. Chromatin was extracted from OCP-induced cells after treating with an EZH1/EZH2 inhibitor and subject to Western blotting with H3CT antibody.

**Additional file 6: Fig. S6.** Schematic representation of ChIPac-qPCR assay.

## Abbreviations

H3NT: histone H3N-terminal tail
H3K27me1: H3 monomethylation at lysine 27
RANK: receptor activator of NF-κB ligand
MMPs: matrix metalloproteinases
OCP: osteoclast precursor
ChIPac: ChIP of acetylated chromatin
TRAP: tartrate-resistant acid phosphatase

## References

[1] Matsuo K, Irie N (2008). Osteoclast-osteoblast communication. Arch Biochem Biophys.

[2] Nakahama K (2010). Cellular communications in bone homeostasis and repair. Cell Mol Life Sci.

[3] Ash P, Loutit JF, Townsend KMS (1980). Osteoclasts derived from haematopoietic stem cells. Nature.

[4] Ikeda K, Takeshita S (2014). Factors and mechanisms involved in the coupling from bone resorption to formation: how osteoclasts talk to osteoblasts. J Bone Metab.

[5] Raggatt LJ, Partridge NC (2010). Cellular and molecular mechanisms of bone remodeling. J Biol Chem.

[6] Nakashima T, Takayanagi H (2011). New regulation mechanisms of osteoclast differentiation. Ann N Y Acad Sci.

[7] Yasuda H, Shima N, Nakagawa N, Yamaguchi K, Kinosaki M, Mochizuki S, Tomoyasu A, Yano K, Goto M, Murakami A (1998). Osteoclast differentiation factor is a ligand for osteoprotegerin/osteoclastogenesis-inhibitory factor and is identical to TRANCE/RANKL. Proc Natl Acad Sci USA..

[8] Teitelbaum SL, Ross FP (2003). Genetic regulation of osteoclast development and function. Nat Rev Genet.

[9] Baron R (2004). Arming the osteoclast. Nat Med.

[10] Ishida N, Hayashi K, Hoshijima M, Ogawa T, Koga S, Miyatake Y, Kumegawa M, Kimura T, Takeya T (2002). Large scale gene expression analysis of osteoclastogenesis in vitro and elucidation of NFAT2 as a key regulator. J Biol Chem.

[11] Tetsuro Y, Jun H, Hiroyuki A, Sakae T (2012). Recent advance in epigenetics—application to the regulation of osteoclast differentiation. Curr Rheumatol Rev.

[12] Vrtacnik P, Marc J, Ostanek B (2014). Epigenetic mechanisms in bone. Clin Chem Lab Med.

[13] Yasui T, Hirose J, Tsutsumi S, Nakamura K, Aburatani H, Tanaka S (2011). Epigenetic regulation of osteoclast differentiation: possible involvement of Jmjd3 in the histone demethylation of Nfatc1. J Bone Miner Res.

[14] Fang C, Qiao Y, Mun SH, Lee MJ, Murata K, Bae S, Zhao B, Park-Min KH, Ivashkiv LB (2016). Cutting edge: EZH2 promotes osteoclastogenesis by epigenetic silencing of the negative regulator IRF8. J Immunol.

[15] Cantley MD, Fairlie DP, Bartold PM, Rainsford KD, Le GT, Lucke AJ, Holding CA, Haynes DR (2011). Inhibitors of histone deacetylases in class I and class II suppress human osteoclasts in vitro. J Cell Physiol.

[16] Birkedal-Hansen H (1995). Proteolytic remodeling of extracellular matrix. Curr Opin Cell Biol.

[17] Papazafiropoulou A, Tentolouris N (2009). Matrix metalloproteinases and cardiovascular diseases. Hippokratia.

[18] Vargova V, Pytliak M, Mechirova V (2012). Matrix metalloproteinases. Exs.

[19] Bergers G, Brekken R, McMahon G, Vu TH, Itoh T, Tamaki K, Tanzawa K, Thorpe P, Itohara S, Werb Z, Hanahan D (2000). Matrix metalloproteinase-9 triggers the angiogenic switch during carcinogenesis. Nat Cell Biol.

[20] Kim K, Punj V, Kim JM, Lee S, Ulmer TS, Lu W, Rice JC, An W (2016). MMP-9 facilitates selective proteolysis of the histone H3 tail at genes necessary for proficient osteoclastogenesis. Genes Dev.

[21] Ferrari KJ, Scelfo A, Jammula S, Cuomo A, Barozzi I, Stutzer A, Fischle W, Bonaldi T, Pasini D (2014). Polycomb-dependent H3K27me1 and H3K27me2 regulate active transcription and enhancer fidelity. Mol Cell.

[22] McCabe MT, Graves AP, Ganji G, Diaz E, Halsey WS, Jiang Y, Smitheman KN, Ott HM, Pappalardi MB, Allen KE (2012). Mutation of A677 in histone methyltransferase EZH2 in human B-cell lymphoma promotes hypertrimethylation of histone H3 on lysine 27 (H3K27). Proc Natl Acad Sci USA.

[23] Sarma K, Margueron R, Ivanov A, Pirrotta V, Reinberg D (2008). Ezh2 requires PHF1 to efficiently catalyze H3 lysine 27 trimethylation in vivo. Mol Cell Biol.

[24] Shen X, Liu Y, Hsu YJ, Fujiwara Y, Kim J, Mao X, Yuan GC, Orkin SH (2008). EZH1 mediates methylation on histone H3 lysine 27 and complements EZH2 in maintaining stem cell identity and executing pluripotency. Mol Cell.

[25] Wu H, Chen X, Xiong J, Li Y, Li H, Ding X, Liu S, Chen S, Gao S, Zhu B (2011). Histone methyltransferase G9a contributes to H3K27 methylation in vivo. Cell Res.

[26] Kubicek S, O’Sullivan RJ, August EM, Hickey ER, Zhang Q, Teodoro ML, Rea S, Mechtler K, Kowalski JA, Homon CA (2007). Reversal of H3K9me2 by a small-molecule inhibitor for the G9a histone methyltransferase. Mol Cell.

[27] Konze KD, Ma A, Li F, Barsyte-Lovejoy D, Parton T, Macnevin CJ, Liu F, Gao C, Huang XP, Kuznetsova E (2013). An orally bioavailable chemical probe of the Lysine Methyltransferases EZH2 and EZH1. ACS Chem Biol.

[28] Antoni C, Vera L, Devel L, Catalani MP, Czarny B, Cassar-Lajeunesse E, Nuti E, Rossello A, Dive V, Stura EA (2013). Crystallization of bi-functional ligand protein complexes. J Struct Biol.

[29] Kozakov D, Hall DR, Xia B, Porter KA, Padhorny D, Yueh C, Beglov D, Vajda S (2017). The ClusPro web server for protein–protein docking. Nat Protoc.

[30] Sengoku T, Yokoyama S (2011). Structural basis for histone H3 Lys 27 demethylation by UTX/KDM6A. Genes Dev.

[31] Del Rizzo PA, Trievel RC (2014). Molecular basis for substrate recognition by lysine methyltransferases and demethylases. Biochim Biophys Acta.

[32] Kaniskan HU, Jin J (2017). Recent progress in developing selective inhibitors of protein methyltransferases. Curr Opin Chem Biol.

[33] Tsuda H, Zhao N, Imai K, Ochiai K, Yang P, Suzuki N (2013). BIX01294 suppresses osteoclast differentiation on mouse macrophage-like Raw264.7 cells. Bosn J Basic Med Sci.

[34] Duncan EM, Muratore-Schroeder TL, Cook RG, Garcia BA, Shabanowitz J, Hunt DF, Allis CD (2008). Cathepsin L proteolytically processes histone H3 during mouse embryonic stem cell differentiation. Cell.

[35] Steiner LA, Schulz VP, Maksimova Y, Wong C, Gallagher PG (2011). Patterns of histone H3 lysine 27 monomethylation and erythroid cell type-specific gene expression. J Biol Chem.

[36] Kim JM, Kim K, Punj V, Liang G, Ulmer TS, Lu W, An W (2015). Linker histone H1.2 establishes chromatin compaction and gene silencing through recognition of H3K27me3. Sci Rep.

