# Supplementary figures and images for: H3K27me1 is essential for MMP-9-dependent H3N-terminal tail proteolysis during osteoclastogenesis
Source: Epigenetics Chromatin. 2018 May 28;11:23. doi: 10.1186/s13072-018-0193-1 (PMC5971420; doi:10.1186/s13072-018-0193-1)

# Additional file 1

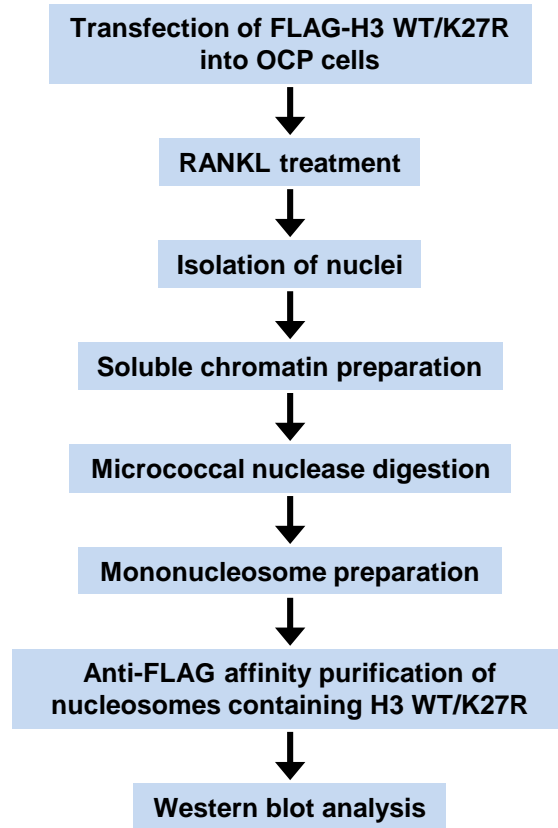

Supplement: Supplementary file 1 — Additional file 1: Fig. S1. Workflow of the purification method used for isolation of ectopic H3 nucleosomes. [file 13072_2018_193_MOESM1_ESM.pdf]

Additional file 2

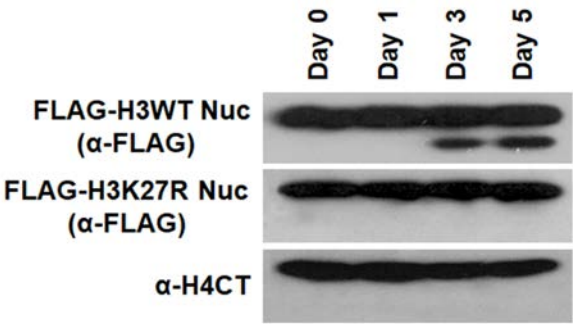

Supplement: Supplementary file 2 — Additional file 2: Fig. S2. Abolishment of osteoclastogenic H3NT proteolysis by H3K27R mutation. Mononucleosomes containing ectopic H3 were purified from OCP-induced cells expressing H3 wild type or K27R mutant with C-terminal FLAG tag as summarized in Additional file 1: Fig. S1 and analyzed by Western blotting with anti-FLAG antibody. [file 13072_2018_193_MOESM2_ESM.pdf]

**a**

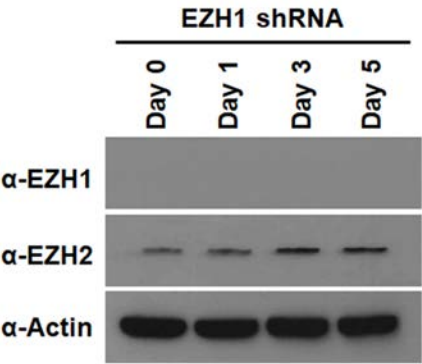

**b**

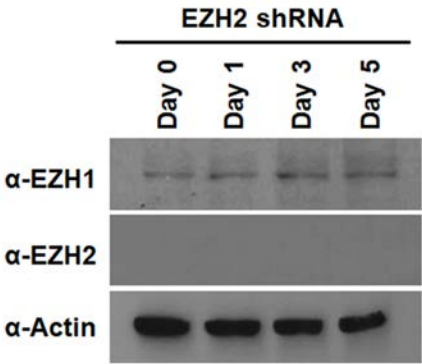

Supplement: Supplementary file 3 — Additional file 3: Fig. S3. Validation of specific knockdown of EZH1 and EZH2. OCP cells were transduced with lentiviral shRNAs targeting EZH1 (a) and EZH2 (b), and knockdown efficiency and specificity were determined by Western blot. [file 13072_2018_193_MOESM3_ESM.pdf]

**a**

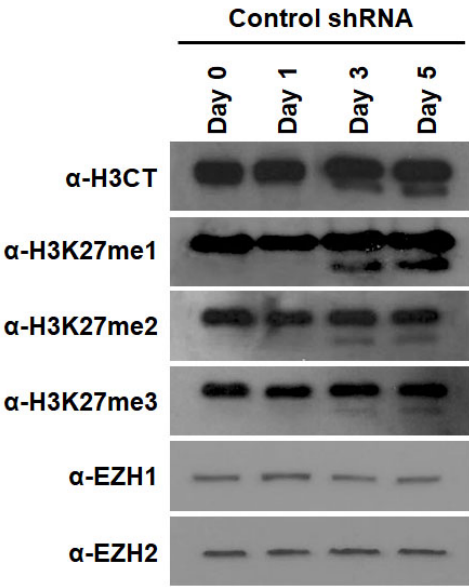

**b**

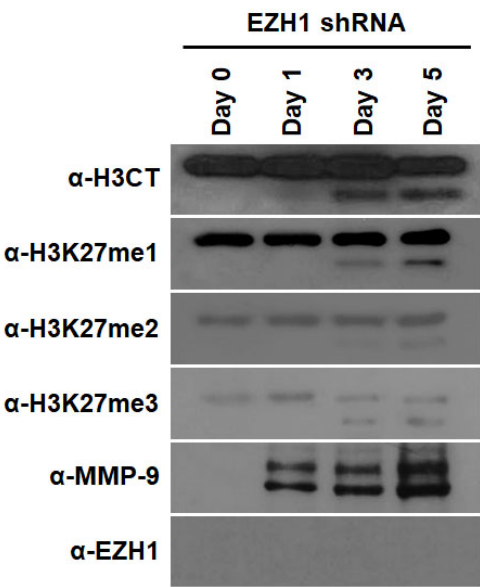

**c**

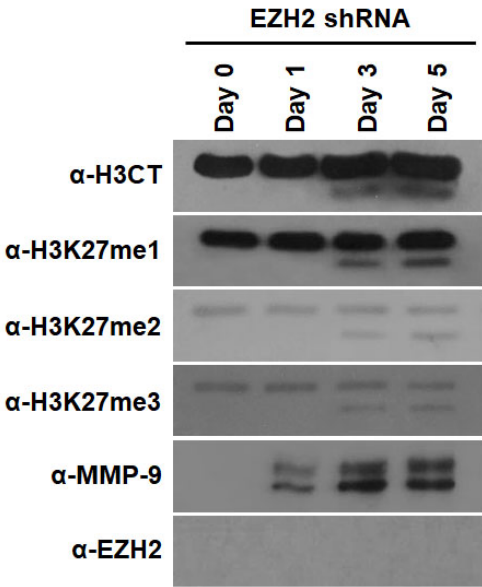

Supplement: Supplementary file 4 — Additional file 4: Fig. S4. Determination of the effects of EZH1/EZH2 knockdown on osteoclastogenic H3NT proteolysis. a Chromatin was purified from mock-depleted, OCP-induced cells, and Western blot analysis for H3NT proteolysis was performed as described in Fig. 2. b As for (a) but using chromatin from EZH1-depleted, OCP-induced cells. c As for (a) but using chromatin from EZH2-depleted, OCP-induced cells. [file 13072_2018_193_MOESM4_ESM.pdf]

Additional file 5

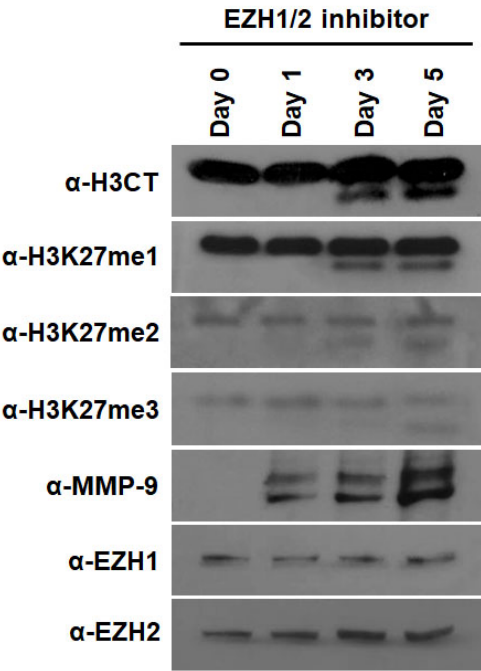

Supplement: Supplementary file 5 — Additional file 5: Fig. S5. Determination of the effects of EZH1/EZH2 inhibitors on osteoclastogenic H3NT proteolysis. Chromatin was extracted from OCP-induced cells after treating with an EZH1/EZH2 inhibitor and subject to Western blotting with H3CT antibody. [file 13072_2018_193_MOESM5_ESM.pdf]

Additional file 6

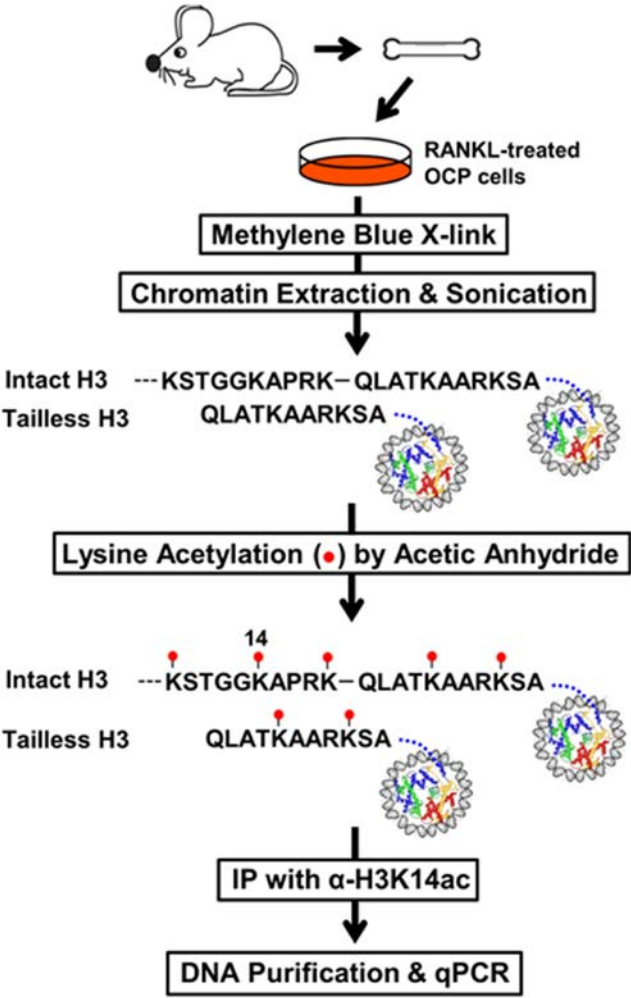

Supplement: Supplementary file 6 — Additional file 6: Fig. S6. Schematic representation of ChIPac-qPCR assay. [file 13072_2018_193_MOESM6_ESM.pdf]
